# Three years of follow-up of otodental syndrome in 3-year-old Chinese boy: a rare case report

**DOI:** 10.1186/s12903-019-0860-z

**Published:** 2019-07-25

**Authors:** Ji-mei Su, Su-juan Zeng, Xiao-wei Ye, Zhi-fang Wu, Xin-wen Huang, Janak L. Pathak

**Affiliations:** 1 Department of Stomatology, Children’s Hospital, Zhejiang University School of Medicine, NO. 3333 Binsheng Road, Hangzhou 310052 Zhejiang Province, People’s Republic of China; 2 Key Laboratory of Oral Medicine, Guangzhou Institute of Oral Disease, Affiliated Stomatology Hospital of Guangzhou Medical University, Guangzhou, 510140 China; 3 Department of Pediatric dentistry, Stomatology Hospital, Zhejiang University School of Medicine, NO 395 Yanan Road, Hangzhou 310006 Zhejiang Province, People’s Republic of China; 4 Department of genetics and metabolism, Children’s Hospital, Zhejiang University School of Medicine, NO. 3333 Binsheng Road, Hangzhou 310052 Zhejiang Province, People’s Republic of China

**Keywords:** Otodental syndrome, Globodontia, Odontoma, Tooth eruption, Sensorineural hearing loss, Enamel hypoplasia, Case report

## Abstract

**Background:**

Otodental syndrome is an exceptionally rare autosomal dominant condition characterized by a delayed eruption of posterior teeth, globodontia, lisping, and sensorineural hearing loss. In this case report, we reported a 3-year-old Chinese boy with the otodental syndrome.

**Case presentation:**

A 3-year-old Chinese boy was referred to our hospital with complaint of no eruption of primary canines and molars. Three years follow-up showed lately erupted bulbous primary canines with hypoplastic enamel spot, globe-shaped primary molars and sensorineural hearing loss at 4 and a half-year-old age. We diagnosed otodental syndrome in the patient’s mother with hearing loss at 16-year-old age. Gene sequencing and analysis of deafness-related genes GJB2, GJB3, SLC26A4, and mtDNA did not reveal any mutation or SNPs in the patient and his mother.

**Conclusions:**

This case report highlights the importance of detailed medical, dental, and family history examination, as well as multi-disciplinary teamwork for diagnosis and treatment of otodental syndrome.

**Electronic supplementary material:**

The online version of this article (10.1186/s12903-019-0860-z) contains supplementary material, which is available to authorized users.

## Background

Otodental syndrome (OMIM 166750) is a rare autosomal dominant condition characterized by grossly enlarged globe-shaped canine and molar teeth (globodontia), and sensorineural hearing loss [1]. Otodental syndrome has been reported only in 12 families so far, including 2 Chinese families [2, 3]. The condition has been reported under various names: familial otodentodysplasia [4], otodental dysplasia [2, 5], globodontia [1, 6, 7] and oculo-oto-dental (ODD) syndrome [8]. Vieira and colleagues reported otodental syndrome associated ocular phenotype and it was named as OOD syndrome [8]. The first case of the otodental syndrome was described in Hungary in a mother and her son by Denes and Csiba in 1969 [9]. In 1976, Witkop et al. named the typical abnormal tooth morphology as globodontia and proposed the concept of the otodental syndrome. Globodontia occurs in both primary and permanent dentition, affecting canine and molar teeth [1]. Globodonta is a striking dental phenotype, characterized by abnormal bulbous enlargement of tooth crown with almost no discernable cusps and grooves, which is both pathognomonic and diagnostic feature of the otodental syndrome [10, 11]. However, primary and permanent incisors are not affected and display normal shape and size [10, 11]. Besides the globodontia, an enamel defect (hypoplasia) is frequently found on the buccal surface of canines. Odontoma is the most commonly seen odontogenic tumor. Odontoma was reported by Beck-Mannagetta et al. [12] and Liu et al. [3] in the posterior maxilla and mandible. Pieces of literature had reported dysmorphic facial features including a long face, full cheek, anteverted nostrils, a long philtrum, and a cleft lip in some patients with otodental syndrome [1, 13, 14]. Bilateral sensorineural hearing deficiency to about 65 dB is found at all frequencies but is more pronounced at about 1000 Hz in these patients. The age of onset of the hearing loss ranges from early childhood to middle age. Moreover, mutations in GJB2, GJB3, SLC26A4, and mtDNA gene are reported as a prominent cause of congenital sensorineural hearing loss [15, 16].

The present case report describes the clinical features of the otodental syndrome, and the eruption time and sequence of posterior teeth through 3 years of follow-up in a 3-year-old Chinese boy. To our knowledge, this is the first report presenting the eruption time and sequence of posterior teeth in a patient with the otodental syndrome.

## Case presentations

**Fig. 1:**
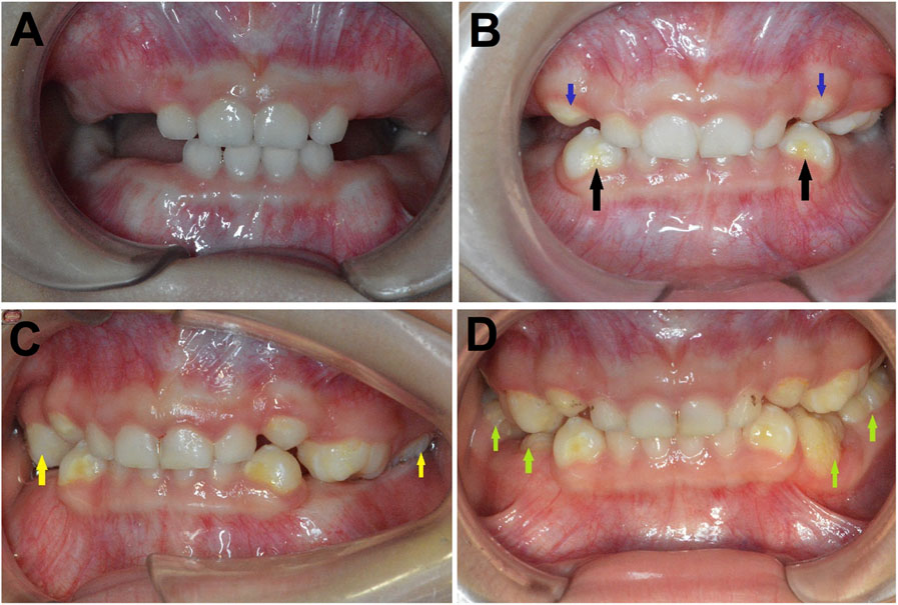
Frontal view of the patient’s of primary dentition. (**a**) Only primary incisors with normal size and shape have erupted at a 3-year-age. (**b**) Canines and left maxillary first primary molar erupted at 4-year-age. Hypomineralized yellow spots on the buccal of mandibular primary canines (black arrows) and a white spots on the buccal of maxillary primary canines (blue arrows) were observed. (**c**) Left maxillary second primary molar and right maxillary first primary molar erupted (yellow arrows) at 5-year-age. (**d**) All primary teeth erupted by a 6-year-age. Green arrows indicate the primary teeth erupted at a 6-year

A 3-year-old Chinese boy was referred to our hospital on 13th July 2015 with a complaint of no eruption of primary canines and molars. His medical and development history revealed that he was born at full term by normal vaginal delivery after an uneventful pregnancy with 50 cm height and 3.3 kg weight. The mother was 29 years old at the time of delivery. The patient was in a normal growth status without short height and developmental malformation. He could hold his head up at 4 months and rollover at 7 months and learn to walk and speak at 1 year. He walked very well but he spoke with a lisp. According to parents, his first baby tooth erupted at 8 months and all of the baby incisors were erupted by 1 and a half-year age. None of canine or molar has erupted until 3-year-old. Parents took the patient to our hospital to find out the reason for the delayed eruption of posterior teeth. Family history indicated that his mother spoke with a lisp and she had hearing loss although her teeth eruption was not delayed. His physical examination revealed normal weight and height compared with the same age children. No abnormality was observed on facial appearance and facial proportions. Intraoral examination revealed that only primary incisors erupted and were normal in size, shape, and color (Fig. 1a). Good oral hygiene with normal overjet and overlap existed. Oral mucosa color and texture were within normal limits. The alveolar ridge of the posterior teeth region was full (Fig. 1a). The parents were informed of the objectives of this research and written informed consent was obtained prior to conducting the study. The study was approved by the Ethical Committee of Children’s hospital, the School of Medicine, Zhejiang University, Hangzhou, China. The investigations were carried out following the rules of the Declaration of Helsinki.

**Fig. 2:**
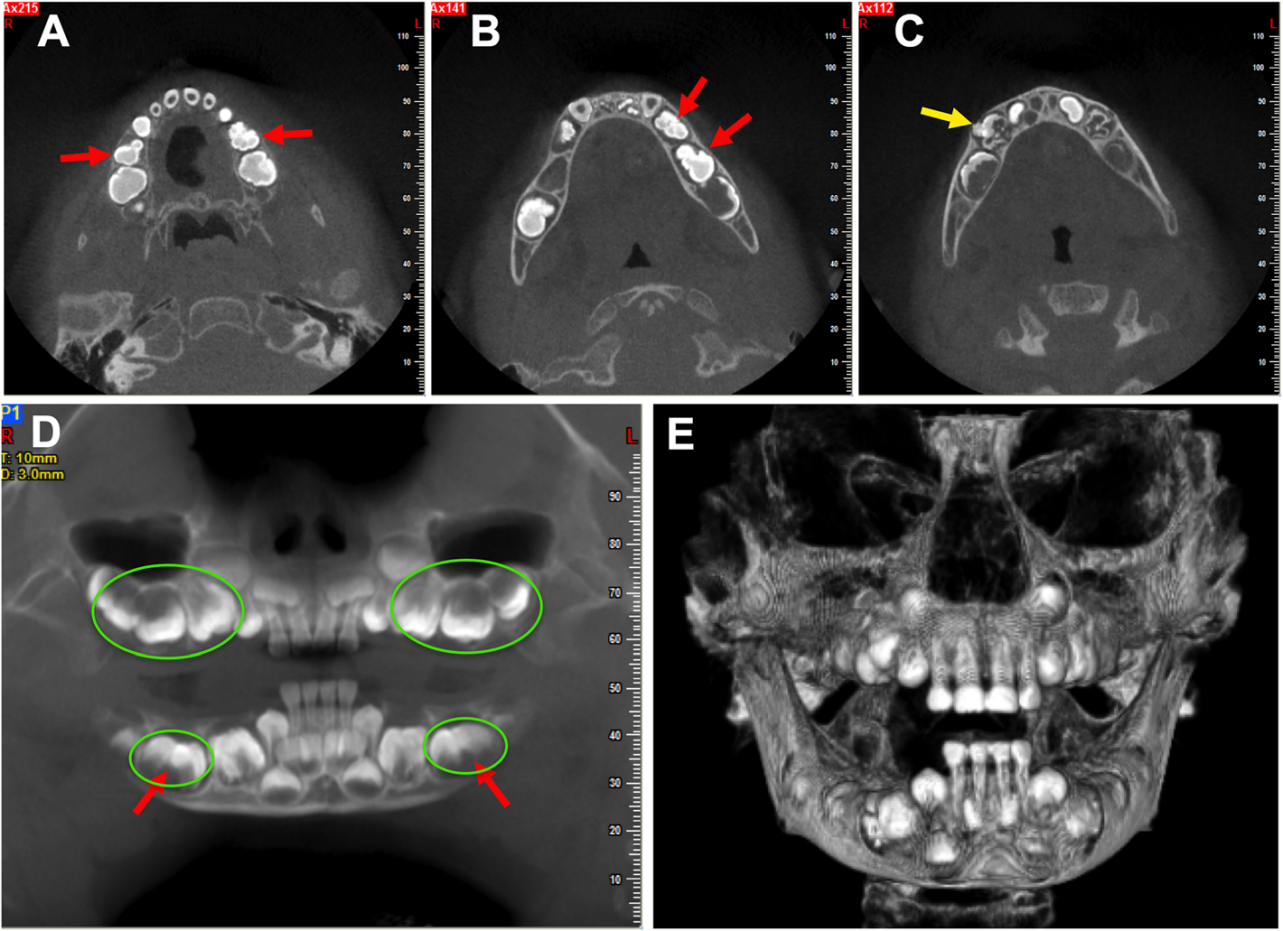
CBCT images of jaws at 3-year-old age. (**a**, **b**) Primary molars teeth are formed by the fusion of smaller and malformed teeth as indicated (red arrows). (**c**) Tooth-like mass was detected in the right mandibular first primary molar region (yellow arrow). (**d**) The crowns of primary canines and globe-shaped primary molars (green circles). Primary molars teeth are formed by the fusion of smaller and malformed teeth (red arrows). (**e**) The CBCT image showed normal craniofacial skeletons

**Fig. 3:**
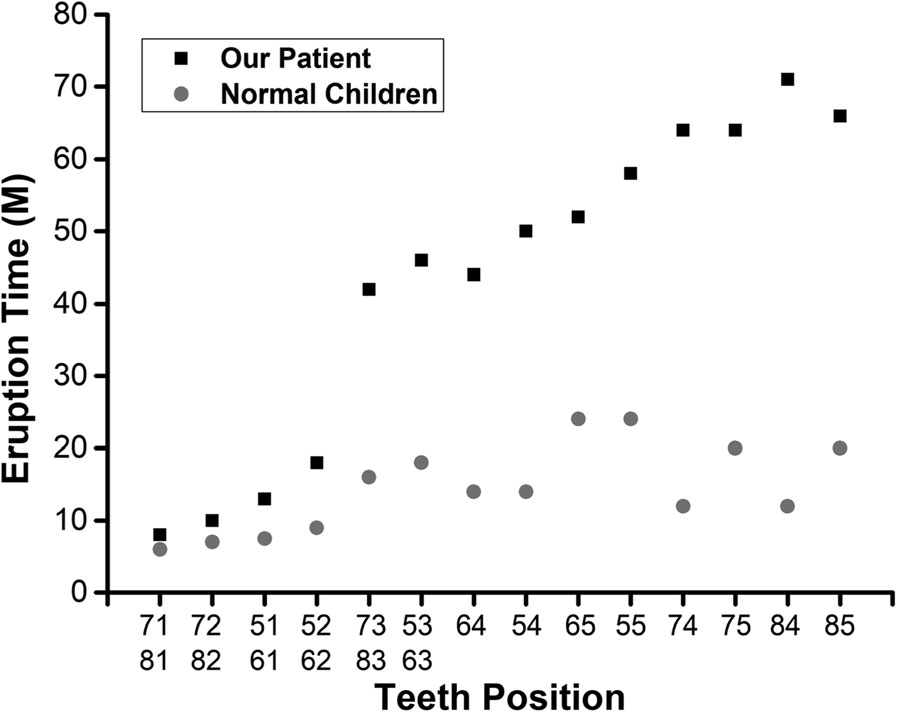
Eruption time of primary teeth in the patient with otodental syndrome compared to that in a normal child. In our patient, Delayed eruption of primary canines and molars was observed. M: months

The patient was uncooperative to take a panoramic radiograph. During panoramic radiograph, the patient should be in stand up position without moving the head position, which is difficult in the case of the pediatric patient. Therefore, we took a cone-beam computed tomography (CBCT) examination under oral sedation (chloral hydrate). The CBCT images revealed that the crowns of primary canines were all bulbous, larger than normal. The premolar area was occupied by macrodontic teeth that appeared to be formed by the fusion of smaller and malformed teeth (Fig. 2a-d). Tooth-like mass was detected in the right mandibular first primary molar region Fig. 2c. Overall craniofacial skeletons were normal (Fig. 2e). The patient did not have any sign of hearing loss at this time. Because of the limits of our knowledge the preliminary diagnosis was malformed canines and molars. The patient was scheduled for semiannual control visits with the prescription of topical fluoride varnish application and hygiene instructions. However, the parents didn’t follow our suggestions. They took him to our hospital once a year. According to his parents’ memory, the left and right mandibular primary canine (tooth#73,83) have erupted at 3-year and a half-old age. Since then the other primary canines and molars have erupted one after the other. The eruption time of every primary tooth, in this case, was compared to that in a normal child [17]. The pattern of delayed teeth eruption in the patient compared to healthy children of the same age is portrayed in Fig. 3.

**Fig. 4:**
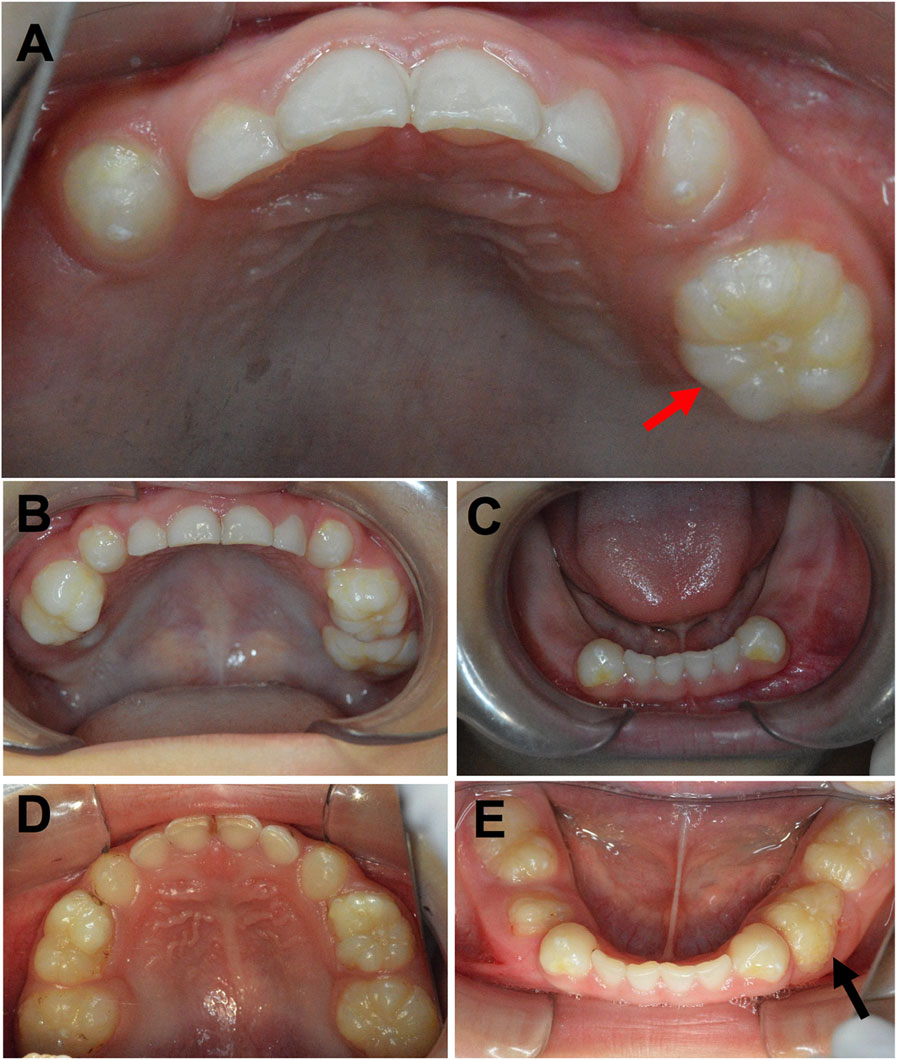
Intraoral views showed abnormal canines and molars at 4-year-old age (**a**), at 5-year-old age (**b**, **c**), and at 6-year-old age (**d**, **e**). The left maxillary first molar was enlarged like a pumpkin (red arrow). The left mandibular first primary molar (#74) was a little mobile with the accumulation of dental plaque on the buccal and lingual gingival margin, and inflamed and enlarged gingiva (black arrow)

The detailed intraoral examination and a panoramic radiograph were taken once a year. His intraoral examination revealed that maxillary and mandibular primary canines and left maxillary first primary molar have erupted at 4-year-old age (1 year after his first visit). The canines appeared larger than normal and displayed bulbous crowns and each had a large bigger cusp on the central (Fig. 1). Each mandibular canine (#73, 83) had a yellow hypoplastic enamel spot on the buccal surface near the gingival margin (Fig. 1). A white hypoplastic enamel spot was present on the buccal surface near the gingival margin of each maxillary canine (#53, 63) (Fig. 1). The left maxillary first primary molar (#64) was gigantic, like a pumpkin and exhibited 7 cusps like projections: mesialbuccal, mesiallingual, buccal, distobuccal, distolingual, lingual and central (Fig. 4a). It presented shallow occlusal grooves extending to the labial, lingual and proximal surfaces (Fig. 4a). The left maxillary second primary molar (#65) and right maxillary first primary molar (#54) have erupted at 5-year-old age (2 years after his first visit). The left maxillary second primary molar (#65) resembled the left maxillary first primary molar (#64) that was enlarged, like a pumpkin. Developmental grooves radiated from the occlusal pit onto the buccal, lingual, and proximal surfaces to the cervical area and divided the crown into lobules of different sizes (Fig. 4b). The right primary first molar (#54) was enlarged and had four major cusps that were more likely two premolars fused from mesial to distal of the dentition arch (Fig. 4b). After 3 years of follow-up (at the patient’s 6-year-old age), all primary molars have erupted although the right mandibular first primary molar was partially erupted (Fig. 4d,e). The right maxillary second molar (#55) resembled the opposite same name tooth (#65) in the shape but the size was larger. The left mandibular first primary molar (#74) was distorted, like a triangle with 3 major rounded cusps. The left mandibular first primary molar (#74) was a little mobile and dental plaque accumulations were found on the buccal and lingual gingival margin, and the gingiva was inflamed and enlarged (Fig. 4e). The mandibular second molars were similarly enlarged with developmental grooves existed dividing the crowns into different sizes of lobules. Caries in maxillary central incisors and right maxillary first primary molar were detected (Fig. 4d).

**Fig. 5:**
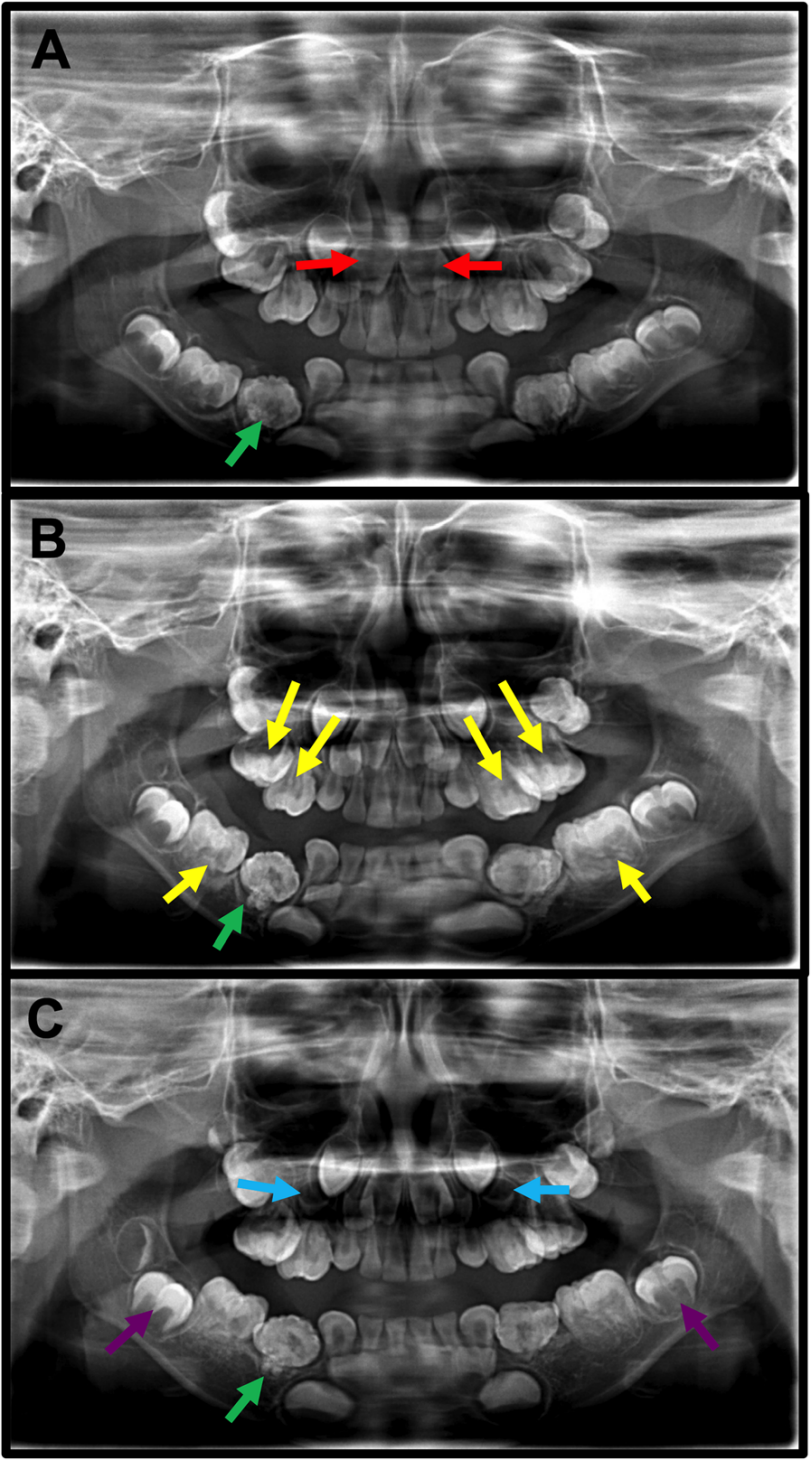
Panoramic picture of dentition at 4-year-old age (**a**), 5-year-old age (**b**), and 6-year-old age (**c**). Red arrows: normal permanent incisor buds; yellow arrows: enlarged and duplicated pulp chamber of primary molar teeth; purple arrows: fused first permanent molar with duplicated pulp chamber; green arrows: odontoma like structure; blue arrows: buds of maxillary first premolar

Radiographs showed the permanent incisors (not yet erupted) to be normal in shape. Primary molars (#54, 55, 64, 65, 75, 85) presented enlarged irregular pulp chambers with trifurcation, creating a macrodontic appearance (Fig. 5a-c). Radiographic appearance suggested these globe-shaped molars be formed by tooth fusion (Fig. 2a, b, d). The first permanent molars were developing within the bone and also had large bulbous fused crown and enlarged pulp chambers with bifurcation (Fig. 5). Roots of primary molars were very short compared to their crowns and developed very slow (Fig. 5). In the right mandibular first primary region, a tooth-like radiopaque mass was appeared extending to the oral cavity from mandibular bone but the roots were not developed (Figs. 5 and 2c). This might be odontoma, however, histological examination is needed for further confirmation. From the panoramic radiograph (at 6-year-old age, Fig. 5c) the buds of maxillary first permanent premolar and permanent canines were detected. The maxillary canines looked like conoid-shaped and smaller than normal maxillary canines. However, the mandibular canines were bulbous and larger.

**Fig. 6:**
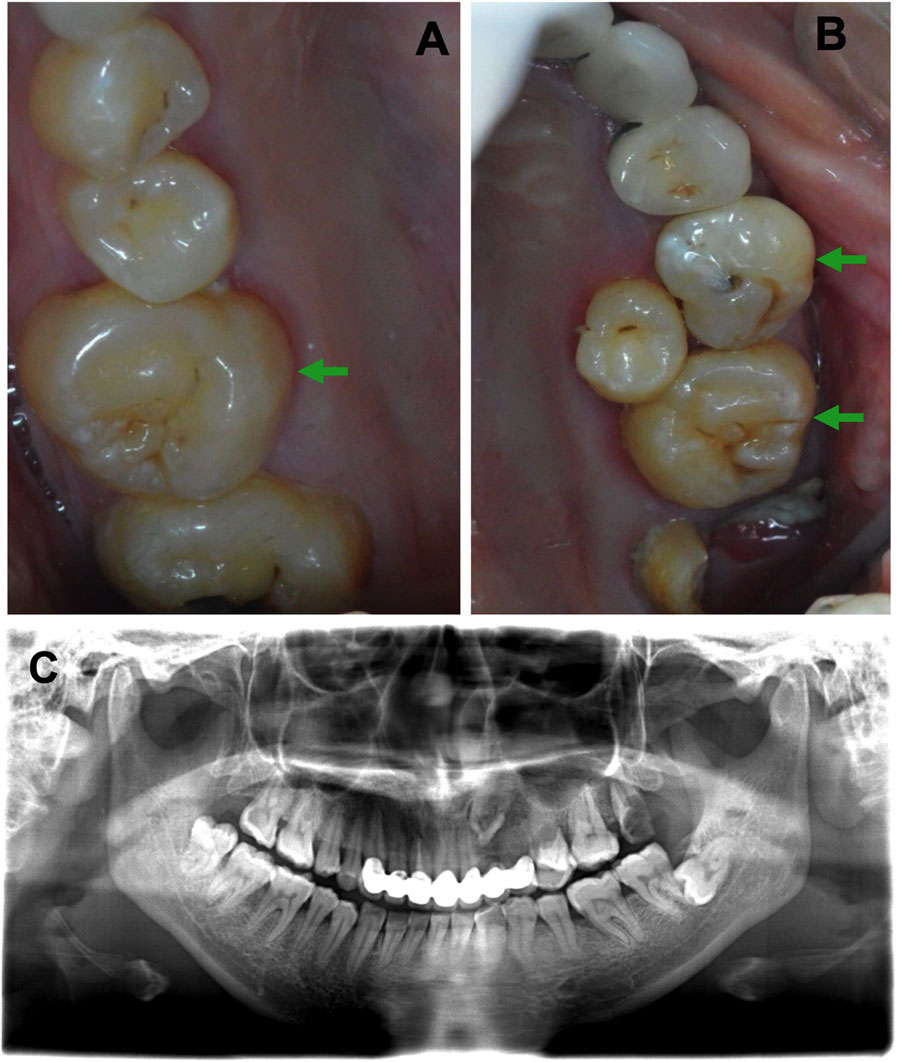
Globe-shaped left maxillary second primary molar and maxillary molars were observed in patient’s mother (**a** and **b**). (**c**) Panoramic radiograph of the patient’s mother with globe-shaped crown in molars

Family history revealed the mother’s hearing loss at 16-year-old age. The audiogram revealed that the mother had a distinct bilateral sensorineural hearing loss of acuity to frequencies above 1000 Hz. Her threshold of the hearing was normal at low frequencies but obviously, drop up to 60 dB at higher frequencies (Additional file 1: Figure S1A and S1B). Parents noticed the hearing loss of the patient at 4 and a half-year-old age. Since the patient was uncooperative in taking the pure tone audiometry testing, the hearing disorder was tested by distortion product otoacoustic emissions (DPOAE), auditory brainstem response (ABR) and auditory steady-state response (ASSR) under oral sedation by chloral hydrate. The acoustic immittance examination revealed type A tympanogram in both ears and DOPAE reports showed refer in both ears and ABR thresholds (2000–4000 Hz click stimuli) were 45 and 90ndBnHL in the left and right ear respectively. ASSR thresholds were 70 and 95 dBHLcg in the left and right ear respectively (Additional file 1: Figure S1C). Otolaryngological examination of this patient confirmed normal auditory structures, temporal bone, and ethmoid air cells. Based on the typical dental phenotype and bilateral high-frequency sensorineural hearing loss, the patient was clinically diagnosed as the otodental syndrome. Mother’s intraoral examination revealed that the left maxillary second primary molar was retained, globe-shaped and larger than normal molar (Fig. 6a and b). The maxillary first permanent molars were bulbous, globe-shaped but the size was normal (Fig. 6a and b). Based on the high-frequency bilateral sensorineural hearing loss, and the malformed primary molar and permanent molars (Fig. 6c), the mother was also clinically diagnosed as the otodental syndrome. The patient’s father was healthy and had no similar clinical signs. Detailed ophthalmic examination of the patient (6-year-old) and his mother (33-year-old) found that neither had evidence of coloboma or other ocular abnormalities. Gene sequencing and analysis of deafness-related genes GJB2, GJB3, SLC26A4, and mtDNA did not reveal any mutation or SNPs in the patient and his mother.

## Discussion

The otodental syndrome is characterized by globodontia and high-frequency sensorineural hearing loss. The first case of the otodental syndrome was described in Hungary in a mother and son by Denes and Csiba in 1969 [9]. After that a British Kindred [8, 18], a girl of Irish extraction [7] and her daughter [19], a family from Brazil [13, 20], a Chinese boy [2], a family of Polish extraction [1], an Austrian family [12], a kindred of Italian extraction followed through six generations [4, 5, 21], a Belgian family [22], an American girl [23], a Korean girl [14] and a Chinese girl [3] has been described in the literature so far. In the case of the otodental syndrome, the dental phenotype is per se diagnostic. Abnormal morphology of the crowns of selected groups of teeth is the most consistent anatomical finding. The molars are gigantic, globe-shaped with absent or shallow fissures and cusps and the canines are bulbous in both dentitions. We found similar abnormalities in primary and permanent teeth, as reported by Santos-Pinto et al. [13] and Levin et al. [4]. However, some reports revealed that the deformities of primary teeth are more severe than for permanent teeth [4].

The incisors were not affected and showed normal in number, shape, and size in both dentitions in this patient as described in the previous case reports [1, 4, 10, 11, 13, 22, 23]. However, Stewart and Kinirous reported conical maxillary lateral incisors in patients with otodental syndrome [7]. Chen et al. observed the presence of extra incisors in the maxillary anterior region and conical supernumerary microdontic teeth on the palatal side of primary maxillary molars [2]. Congenitally absent primary incisors were reported in a Chinese girl with otodental syndrome [3]. A significant delay in eruption of the primary and permanent dentition, especially in the lateral sectors had been reported in most of otodental syndrome cases [1, 20]. Santos-Pinto et al. reported a one-year delay in eruption of primary canines and molar teeth [13]. In our study, primary canines and molars eruption time was delayed about 2 to 3 years. During the three-year follow-up period, primary canines and molars were erupted one after the other (as shown in Fig. 3). The eruption of primary dentition was almost completed at a 6-year-old age. This is the first study to describe the eruption time and sequence of primary canines and molars in the patient with otodental syndrome in detail. Small premolar or absence of premolars has been frequently reported is the otodental syndrome cases [2, 4–7, 10–13, 20–22]. In the present case report, the germs of mandibular premolars and the maxillary second premolars were not detected in the panoramic film at a 6-year-old age. Therefore, we could not confirm the absence of premolars due to the delayed development nature of both dentitions and we need more time to wait and observe. Similarly, Cehreli et al. reported a case, with undetected premolar buds in either jaw at a 6-year-old girl [23].

Odontoma occurs mainly in children and young adults, especially during their second decade of life [24]. Complex odontoma tends to occur in the posterior part of the jaws and consists of disorganized masses of hard and soft dental tissues with no morphological resemblance to normal teeth [25]. Beck-Mannagetta et al. reported odontoma in an otodental syndrome patient in 1984 [12]. Liu et al. reported odontoma as a cause of the pain and the cutaneous sinus tracts in a patient with otodental syndrome [3]. In the present case report, tooth-like radiopaque mass extending to the oral cavity from mandibular bone was observed in the right mandibular first primary region. However, further follow up, or histological examination is required to rule out the possibility of odontoma.

In otodental syndrome, both the enamel hypoplasia and the deep fissures on bulbous molars are susceptible to dental caries [10]. In our study, oral hygiene was fair as shown in Fig. 1, which needs a regular filling treatment to restore most of the carious lesions. However, endodontic therapy could be quite complex and challenging due to the duplicated pulp canals in the affected posterior teeth. Mesaros et al. have presented a case with the complexity of endodontic treatment in a permanent tooth with globodontia [26]. The authors emphasized the challenges of endodontic treatment and propensity of globodontic teeth to develop endodontic-periodontic lesions due to their aberrant coronal and pulpal morphology. Thus, preventive procedures (i.e. topical fluorides, sealants) should be carried out on a regular basis in patients with the otodental syndrome. Moreover, due to the lack of normal permanent premolars and molars, in the future, the patient might have an occlusion problem. A pediatric dentist should decide the suitable treatment approach to correct such an occlusion problem.

The otodental syndrome was found to show variable expressivity. Dental abnormalities and hearing loss usually coexist in the patients. However, it was found that some patients develop only sensorineural hearing loss, while others show dental abnormalities without hearing loss [1, 2, 11, 20]. In general, sensorineural hearing loss (hearing threshold greater than 25 dB in one or both ears) is more pronounced at frequencies of about 1000 Hz. The age of onset of hearing loss varies from early childhood to middle age. However, the first symptoms of hearing loss usually occur before the 20-year-old age. Regardless of the age of onset, hearing loss is progressive, but it usually plateaus by the fourth decade [1, 10, 11, 21]. In the present case, hearing loss was found at 4-year-old age, but the mother’s hearing loss was found at her 16-year-old age. In our study, gene sequencing and analysis of deafness-related genes GJB2, GJB3, SLC26A4, and mtDNA did not reveal any mutation or SNPs in the patient and his mother.

## Conclusions

Globodontia, enamel hypoplasia, odontoma, and delayed eruption of primary canines and molars were the main dental phenotype of our patient. Globodontia is a diagnostic feature of the otodental syndrome, which often provides the path to the discovery of the associated hearing loss. This case report provides new information regarding the eruption time of every primary canines and molar teeth in this patient compared to the same age healthy children. This would make dentists more familiar with the diagnosis and management of the otodental syndrome. This study highlights the importance of taking a detailed medical, dental, and family history for diagnosis and treatment of this rare disease. An interdisciplinary approach including regular follow up, scheduled tooth extraction, eventually, orthodontic treatment, eye examination, hearing test, and hearing aid are recommended for diagnosis and treatment of the otodental syndrome.

## Additional file

Additional file 1: **Figure S1** Pure tone audiogram of the patient’s mother (A and B). Air conduction (X, left ear; right ear) and bone conduction (>, left ear; <, right ear) demonstrating high-frequency sensorineural hearing loss. (C) ASSR Audiogram of the patient (O, right ear; X, left ear). (DOCX 371 kb)

## Data Availability

The clinical data used during the current study are available from the corresponding author on reasonable request. If clinical data are shared, they will be anonymized.

## Abbreviations

CBCT: Cone beam computed tomography
ODD: Oculo-oto-dental syndrome
