# Supplementary material for: Three years of follow-up of otodental syndrome in 3-year-old Chinese boy: a rare case report
Source: BMC Oral Health. 2019 Jul 25;19:164. doi: 10.1186/s12903-019-0860-z (PMC6659250; doi:10.1186/s12903-019-0860-z)
Supplement: Supplementary file 1 — Figure S1 Pure tone audiogram of the patient’s mother (A and B). Air conduction (X, left ear; right ear) and bone conduction (>, left ear; <, right ear) demonstrating high-frequency sensorineural hearing loss. (C) ASSR Audiogram of the patient (O, right ear; X, left ear). (DOCX 371 kb) [file 12903_2019_860_MOESM1_ESM.docx]

**Supplementary Data**

**Title: Three Years of Follow-up of Otodental Syndrome in 3-year-old Chinese boy: A Rare Case Report**

**Authors:** Ji-mei Su, Su-juan Zeng, Xiao-wei Ye, Zhi-fang Wu, Xin-wen Huang, Janak L. Pathak

**Supplementary Figure 1.** Pure tone audiogram of the patient’s mother (A and B). Air conduction (X, left ear; right ear) and bone conduction (>, left ear; <, right ear) demonstrating high-frequency sensorineural hearing loss. (C) ASSR Audiogram of the patient (O, right ear; X, left ear).


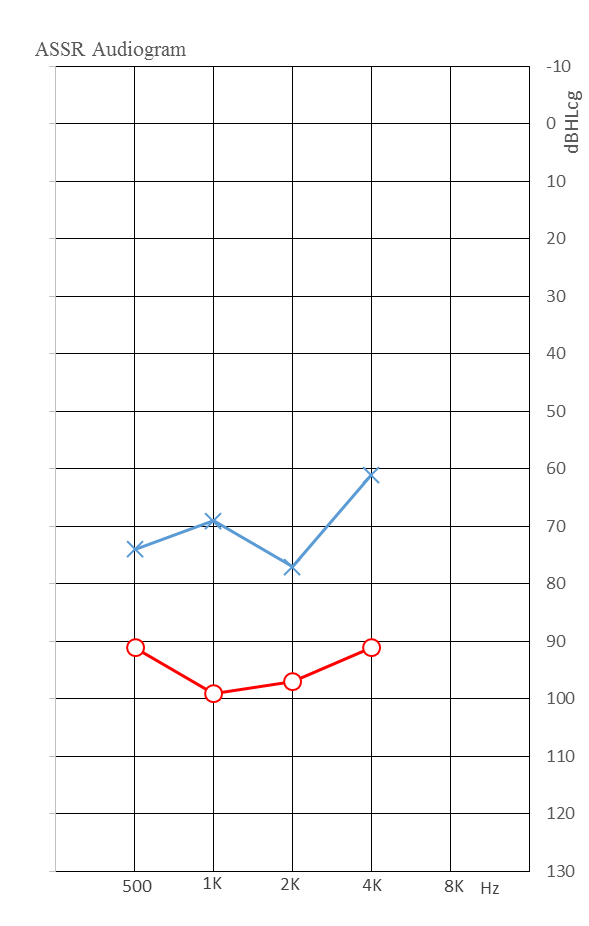


**C**

**Frequency (Hz)**


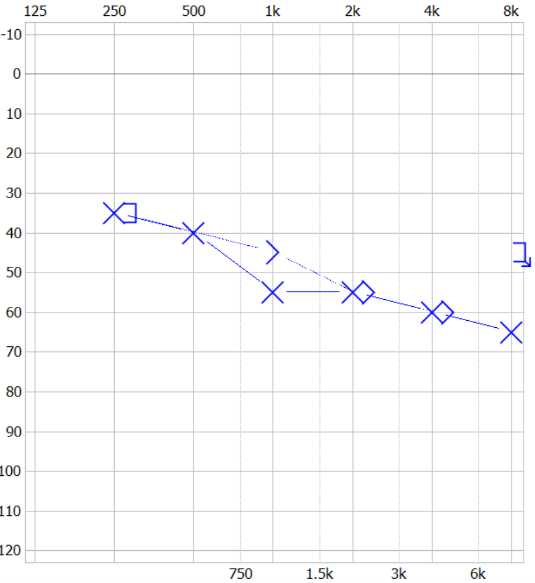


**Frequency (Hz)**

**Hearing Level (dB)**


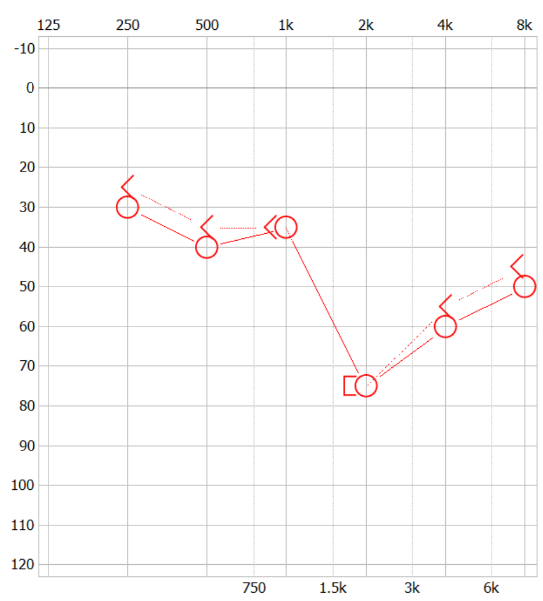


**A**

**B**

**Hearing Level (dB)**
